# Catch the drift: Depressive symptoms track neural response during more efficient decision-making for negative self-referents

**DOI:** 10.1016/j.jadr.2023.100593

**Published:** 2023-05-19

**Authors:** Peter J. Castagna, Allison C. Waters, Elizabeth V. Edgar, Rotem Budagzad-Jacobson, Michael J. Crowley

**Affiliations:** a Yale Child Study Center, Yale School of Medicine, New Haven, CT, United States; b Nash Family Center for Advanced Circuit Therapeutics, Icahn School of Medicine at Mount Sinai, New York, NY, United States

**Keywords:** Self-referential processing, Adolescence, Social anxiety, Depression, Drift diffusion

## Abstract

**Background:**

Adolescence is a time of heightened risk for developing depression and also a critical period for the development and integration of self-identity. Despite this, the relation between the neurophysiological correlates of self-referential processing and major depressive symptoms in youth is not well understood. Here, we leverage computational modeling of the self-referential encoding task (SRET) to identify behavioral moderators of the association between the posterior late positive potential (LPP), an event-related potential associated with emotion regulation, and youth self-reported symptoms of depression. Specifically, within a drift-diffusion framework, we evaluated whether the association between the posterior LPP and youth symptoms of major depression was moderated by drift rate, a parameter reflecting processing efficiency during self-evaluative decisions.

**Methods:**

A sample of 106 adolescents, aged 12 to 17 (53% male; *M*_age_ = 14.49, *SD* = 1.70), completed the SRET with concurrent high-density electroencephalography and self-report measures of depression and anxiety.

**Results:**

Findings indicated a significant moderation: for youth showing greater processing efficiency (drift rate) when responding to negative compared to positive words, larger posterior LPPs predicted greater depressive symptom severity.

**Limitations:**

We relied on a community sample and our study was cross-sectional in nature. Future longitudinal work with clinically depressed youth would be beneficial.

**Conclusions:**

Our results suggest a neurobehavioral model of adolescent depression wherein efficient processing of negative information co-occurs with increased demands on affective self-regulation. Our findings also have clinical relevance; youth’s neurophysiological response (posterior LPP) and performance during the SRET may serve as a novel target for tracking treatment-related changes in one’s self-identity.

## 1. Introduction

There is a sharp increase in the point prevalence of depression from childhood (1–3%; Costello et al., 2006) to adolescence (5–7%; Costello et al., 2006; Kessler et al., 2005). Adolescence is also a critical period for the development and integration of self-identity (e.g., Arain et al., 2013; Blakemore, 2008; Erikson, 1956; Wilson, 1987). Major depression is characterized by chronic low self-competence, self-worth, and poor self-concept (Harter and Jackson, 1993; King et al., 1993; Lewinsohn et al., 1998a, 1998b). Indeed, negative biases in self-referential processing serves as a cognitive vulnerability for developing major depression (Beck, 2008; Beevers et al., 2019; Dainer-Best et al., 2018, 2018; Dainer-Best et al., 2017; Gotlib and Joormann, 2010). Self-referential processing is conceptualized as a set of latent mechanisms that gives rise to individual differences in the processing of both positive and negative self-descriptors during self-reflection (Dainer-Best et al., 2018), most often examined through the Self-Referential Encoding Task.

### 1.1. Self-Referential processing

The self-referential encoding task (SRET) has been widely used to study self-referential processing of emotional stimuli, and therefore, is a suitable candidate for a task assessing self-concept, an important phenotype of major depression (Beck, 2008; Beevers et al., 2019) and social anxiety (Clark and Wells, 1995; Dainer-Best et al., 2018; Wells et al., 1995). Participants report if negative and positive adjectives (e.g., stupid; smart) are self-descriptive (e.g., “Yes” or “No”; Derry and Kuiper, 1981). The SRET is an affective decision-making task in which participants make forced-choice responses concerning whether a series of positive and negative adjectives are descriptive of them or not (Dainer-Best et al., 2018). The SRET is an affective decision-making take where participants make forced-choice responses as to whether a series of positive and negative adjectives are self-descriptive (Dainer-Best et al., 2018). Put differently, participants endorse or deny positive/negative self-descriptors consistent with their self-concept of identity.

Past research utilizing the SRET has found that as an individual’s depression symptom severity increases they tend to classify negative words as self-descriptive more easily (Dainer-Best et al., 2018). In fact, depression symptoms have been found to explain between 34% to 45% of the variance in SRET negative-adjective performance (Beevers et al., 2019). Moreover, a recent meta-analysis confirmed depressed individuals recall and endorse fewer self-referential positive than negative words, than nondepressed individuals (Collins and Winer, 2022). The SRET has been studied with event-related brain potentials (ERPs) and computational modeling, further unpacking the neural and behavioral processes underlying self-relevant decisions. A novel innovation is to bring ERPs and computational modeling methods together in the same investigation; no studies to-date have examined the relation between the neural correlates of self-referential processing and depressive symptoms from a drift diffusion framework in youth.

### 1.2. Late positive potential (LPP)

Electroencephalography (EEG) reveals ERPs with excellent temporal resolution making them well-suited for studying emotional processing. The late positive potential (LPP) is a positive-going deflection that becomes visible approximately 400 milliseconds after stimulus onset, with the early portion of the LPP occurring between 400 and 1000 ms, and is pronounced at centro-parietal recording sites (MacNamara et al., 2009). The LPP is of particular interest in current study as the regulatory effects on the LPP may provide a viable marker of developmental maturation in emotion regulation (MacNamara et al., 2022). For instance, the LPP component is larger for emotional (i.e., pictures, faces and words) compared to neutral stimuli and is sensitive to level of arousal (Cuthbert et al., 2000; Hajcak and Foti, 2020). Further, the LLP in response to emotional stimuli serves as a potential neurophysiological indicator of depressive symptomatology (Dickey et al., 2021; MacNamara et al., 2016), and there is a significant literature indicating that an altered LPP response to emotional stimuli is associated with internalizing symptoms across the lifespan (Bunford et al., 2017; Dickey et al., 2021; Foti et al., 2010; MacNamara et al., 2022; MacNamara et al., 2016; McLean et al., 2020). Importantly, the LPP can be reliably assessed across development as an index of sustained attention towards motivationally salient stimuli (Hua et al., 2014; Kujawa et al., 2013). To remain consistent and to facilitate comparison with previous research examining the LPP in response to SRET, the current study will (Auerbach et al., 2016, 2015; Speed et al., 2016), the current study will focus on the posterior LPP.

In regards to self-referential processing in adolescence, compared to healthy controls, there is some research indicating that depressed (Auerbach et al., 2015) and at-risk youth (Speed et al., 2016) have a greater posterior LPP response to negative (relative to positive) self-descriptions during the SRET (Auerbach et al., 2015; Speed et al., 2016). Although limited, research utilizing the SRET typically functioning youth indicates that there is little difference in the amplitude of the posterior LPP across exposure to positive and negative adjectives (Auerbach et al., 2016). Importantly, research indicates that the posterior LPP response may be differentially related to symptoms of depression and anxiety (MacNamara et al., 2016; McLean et al., 2020; Sandre et al., 2019; Weinberg and Sandre, 2018). Despite the potential confounding effect, comorbid social anxiety symptoms are not always taken into consideration (c.f., Castagna et al., 2022; Kessel et al., 2017; MacNamara et al., 2016).

In sum, negative biases in self-referential processing are a cognitive vulnerability for developing major depression (Beck, 2008; Beevers et al., 2019; Dainer-Best et al., 2018, 2018; Dainer-Best et al., 2017; Gotlib and Joormann, 2010). The posterior LPP is a neural response to emotional stimuli that serves as a potential neurophysiological indicator of major depressive symptomatology (Dickey et al., 2021; MacNamara et al., 2016); a perturbed posterior LPP in response to emotional stimuli is associated with internalizing symptoms across the lifespan (Bunford et al., 2017; Dickey et al., 2021; Foti et al., 2010; MacNamara et al., 2022; MacNamara et al., 2016; McLean et al., 2020). Despite this, the relation between the self-referential posterior LPP and symptoms of major depression in youth is not well-understood.

### 1.3. Drift diffusion model (DDM)

Computational modeling methods have advantages when attempting to elucidate a mechanistic understanding of depression symptoms (Gibbs-Dean et al., 2023). In contrast to task-based metrics of average performance (e.g., reaction time), computational models of behavior can provide a mechanistic understanding through systematic decomposition of behavior into its constituent latent processes. Linking the cognitive processes underlying self-referential processing can further our understanding of their relationship with symptoms of depression in youth (Beevers et al., 2019). Specifically, the drift diffusion model (DDM) is a class of sequential sampling process models that identify underlying cognitive mechanisms of decisions by leveraging intraindividual variability across experimental trials and between task conditions (Ratcliff, 1978, 1985; Ratcliff and Childers, 2015; Ratcliff and McKoon, 2008; Ratcliff and Rouder, 1998; Ratcliff and Tuerlinckx, 2002; Stone, 1960). Distributions of response times and decisions made during the task serve as model inputs and results in DDM latent parameters are thought to reflect cognitive components of decision-making. Previously, we found support for the utility of modeling the SRET; the rate of evidence accumulation (i.e., drift rate) during negative self-referential processing was related to social anxiety and depression symptomatology above-and-beyond mean task performance (Castagna et al., 2022). However, no studies to-date have examined the relation between the neural correlates of self-referential processing and depressive symptoms from a drift diffusion framework in youth.

The drift rate, response threshold, and starting point bias DDM parameters are of particular importance when examining self-referential decision-making. The drift rate can be interpreted as a measure of subjective task difficulty. In the SRET, a drift rate parameter estimate reflects the efficiency of the cognitive processes involved in deciding whether a negative or positive adjective is self-descriptive (i.e., processing efficiency). Drift rate has been found to be sensitive to changes in perceptual, memory, value, and affective features of a decision (Nayak et al., 2019; van Vugt et al., 2012; Vugt et al., 2019). In the context of the SRET, an individual with high levels of depression may be more likely to have a smaller drift rate (i.e., less efficient in self-referential processing) potentially reflecting less confident in their sense of self.

The decision threshold parameter reflects the speed-accuracy tradeoff; larger values of the parameter lead to more accurate but slower choices. More specifically, the decision threshold captures to the amount of information an individual required prior to making a decision. Thus, larger threshold separation values reflect a more conservative decision style (Voss et al., 2004) and has been useful when modeling brain dynamics, and emotional decision-making (Forstmann et al., 2008; Mansfield et al., 2011; Nayak et al., 2019). In terms of the SRET, decision threshold reflects the amount of evidence needed to accumulate to decide whether a negative or positive adjective is self-descriptive. For example, high levels of depression may be associated with a larger decision threshold (i.e., more conservative decision style), as they are cautious in how they describe themselves due to fear providing the “wrong” answer.

The starting point bias parameter reflects a preference for one response (e.g., “yes”) over the alternative (e.g., “no”). The preferred option requires less evidence to accumulate while much more evidence is needed to reach the alterative option. During the SRET, the bias parameter governs one’s preference for responding yes/no irrespective of the self-descriptive adjective. An example in the context of the SRET, depression may be associated with a bias towards responding “no” (and therefore, requiring much more evidence to accumulate to respond “yes” for any given adjective) due to feeling too self-conscious to endorse any given positive/negative self-descriptor (Hitchcock et al., 2022).

An assumption of the DDM is that the total choice response time includes a decision and a nondecision portion. While aspects of the decision time are reflected in the three aforementioned parameters, the nondecision time parameter is thought to capture time spent on perceptual and motor processes. While important for modeling purposes, the nondecision time was not the focus of the present study.

### 1.4. Current study

The current study investigated whether the magnitude of the relationship between youth neural response to positive and negative self-descriptors during SRET performance and self-reported depressive symptoms vary as a function of self-referential processing efficiency (i.e., drift rate). We leverage computational modeling of the SRET (Beevers et al., 2019; Dainer-Best et al., 2018; Dainer-Best et al., 2017; Hsu et al., 2020), to evaluate DDM parameters of self-referential processing, posterior LPP responsivity, and youth self-reported symptoms of depressive symptoms. Our goal was to evaluate a possible moderating effect of SRET performance from a drift-diffusion framework on the relation between the posterior LPP during the SRET and depression symptom severity, while controlling for the presence of social anxiety. Theorizing that processing efficiency during self-evaluative decision--making is a characteristic of adolescent depression, we hypothesize that the drift rate parameter will significantly moderate the relation between the posterior LPP neural response and youth depressive symptoms.

## 2. Methods

### 2.1. Participants & procedure

After the removal of three participants due to a large amount of missing demographic/self-report data, the final sample consisted of 106 adolescents (*n*_male_ = 56, *n*_female_ = 50, *M*_age_ = 14.49, *SD* = 1.70). Parents predominantly identified youth as White (*n* = 81, 76.4%), followed by Black (*n* = 9, 8.5%), Hispanic/Latin (*n* = 6, 5.7%), and Asian (*n* = 6, 5.7%). Four (3.8%) participants identified as other or unknown racial/ethnic origins. The majority of the participants self-reported that they were right-handed (i.e., 95.1%). All youth had corrected-to-normal vision and were fluent English speakers. Participants were recruited through a mass mailing list targeting New Haven, CT and the surrounding towns. None of the adolescents participating in the study had a history of traumatic brain injury with loss of consciousness nor a current diagnosis of a psychiatric disorder.

Youth were recruited as part of a large-scale study that used multiple paradigms, including the SRET with concurrent electroencephalography (EEG) (Ke et al., 2020, 2018). The current study is a re-analysis of a previously published study on the SRET that did not apply computational modeling (Ke et al., 2020). In the present cross-sectional study, parents and youth completed self-reported questionnaires in different rooms. Demographic information (e.g., sex, age, ethnicity, and socio-economic status) about the youth and their families was obtained from the parent. Adolescents were compensated $80 USD for participation and their parent or guardian received $10. Parents provided written informed consent and adolescents provided written informed assent following procedures approved by the Yale School of Medicine Human Investigation Committee.

### 2.2. Measures

#### 2.2.1. The self-referential encoding task [SRET]

**Fig. 1.**
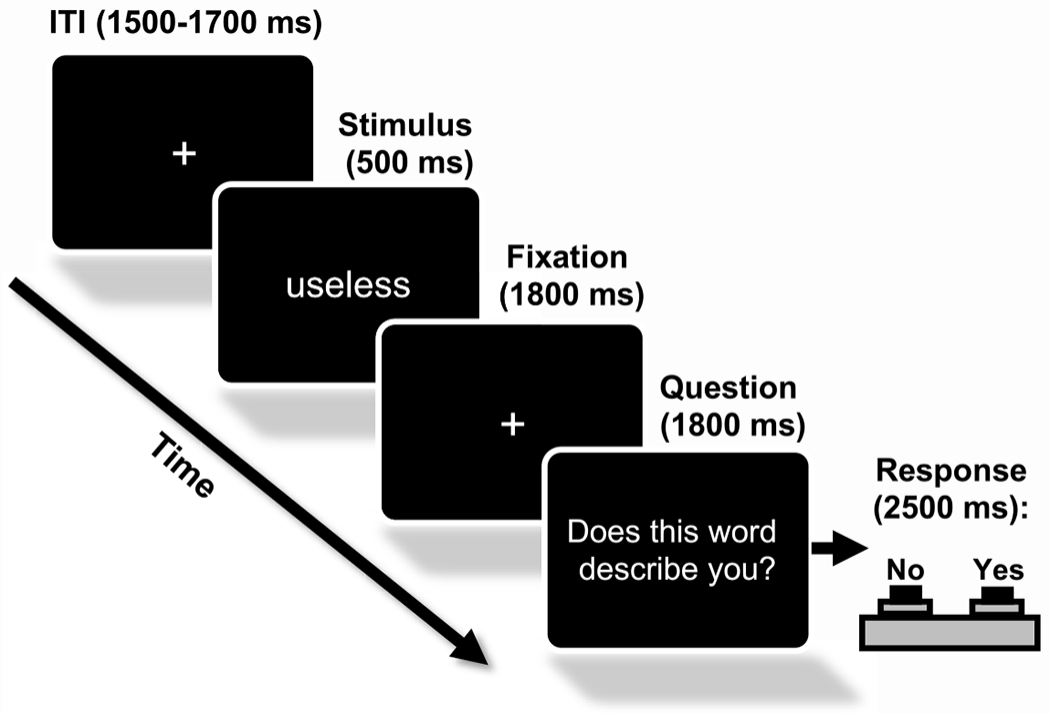
Schematic of the self-referential encoding task used in the current study (not to scale).

The SRET required participants to read a single-word adjective (e.g., “gloomy”, “joyful”) and indicate whether the word was self-descriptive or not self-descriptive. The Oregon Self-Concept inventory (OSCI-II; Tucker et al., 2003) and previous studies with the SRET (Auerbach et al., 2015; Waters and Tucker, 2013) were used to create the 104-word list (see Appendix A). The 52 positive and 52 negative adjectives were balanced for number of letters and reading level. Prior to the administration of the SRET, participants completed three affectively neutral words (e.g., “tall”, “boy”) in the first block and asked if they understood the task to confirm that they understood the instructions before completing the SRET task. For each trial in the second block, the stimulus (i.e., single word adjective) was presented for 500 ms, which was followed by a fixation cross (1800 ms), and then the question, “Does this word describe you?” to which participants responded by quickly pressing the button corresponding to either “yes” or “no”. The text “too slow” was presented if a response was not recorded after 2500 ms. The inter-trial interval (fixation cross) was jittered between 1500 and 1700 ms (see Fig. 1). The average administration time was 10.4 (SD = 2.64) minutes. Participants completed all 104 trials presented in a pseudorandom order with no more than two stimuli of the same valence presented in succession. Stimuli were presented using E-Prime 2.0 (PST, Sharpsburg, PA, USA).

#### 2.2.2. Children’s depression inventory [CDI]

The CDI is a 27-item self-report questionnaire used to assess depressive symptoms in children and adolescents (Kovacs and Preiss, 1992). Items on the CDI address a number of factors, including negative mood, interpersonal problems, ineffectiveness, anhedonia, and negative self-esteem. The reliability and validity of the CDI has been measured extensively and found to be adequate in clinical and non-clinical samples of children and adolescents (e.g., Saylor et al., 1984). A meta-analysis of 283 studies found a Cronbach’s alpha point estimate of 0.84 (Sun and Wang, 2015). In this study, the internal consistency was found to be good (Cronbach’s α = 0.84) for the CDI total-score.

#### 2.2.3. Multidimensional anxiety scale for children [MASC]

The MASC (March 1998) is a 45-item child self-report questionnaire for symptoms of anxiety. Total scores range from 0 to 120, with higher scores indicating greater childhood anxiety. The four empirically derived factor index scores are Social Anxiety, Separation Anxiety, Harm Avoidance, and Physical Symptoms. The MASC has shown good internal consistency ratings from 0.70 to 0.83 and Cronbach’s alpha ranging from 0.74 to 0.85 (March 1998). The MASC has been found to be a clinically useful measure to discriminate between anxious and depressed pediatric patients (Rynn et al., 2006). Here, the internal consistency was found to be good (Cronbach’s α = 0.86) for the MASC Social Anxiety subscale.

### 2.3. EEG data acquisition

During the study, adolescents were seated in a dimly lit, sound-attenuated room 24 inches away from a 19-inch LCD monitor. NetStation (Version 4.5) with a high-impendence amplifier (Series 300 Amplifier) using 128-channel HydroCel gel nets (Magstim-EGI, Eugene, OR) was used to collect the high-density EEG data. Data were recorded at 250 Hz sampling rate with a 0.1 Hz high-pass, and 100 Hz low-pass frequency band filter. Electrode Cz was used as the reference and again referenced offline to the average of all electrodes. Electrode impedances were kept below 50 kΩ after net placement.

### 2.4. Data-Analysis approach

#### 2.4.1. EEG pre-processing

EEG data were processed using NetStation 4.5 (Magstim-EGI, Eugene, OR). A first-order 0.1 Hz low-pass followed by a 30 Hz high-pass filter was applied to the continuous EEG data to reduce noise artifacts. Eprime word-onset tags were used to identify event-related epochs 100 ms pre- and 2000 ms post-stimulus onset. NetStation Ocular Artifact Removal Tool was used to identify (eye channel amplitudes exceeding 150μV) and correct eye blinks and eye movements. Bad channels were defined as having more than 40% of the segment data points exceeding 200 μV and removed. Trials with more than 10 bad channels were excluded from further analysis steps and replaced by surrounding channels through spherical spline interpolation (<10%). Number of bad trials was unrelated to participants CDI total score (*r* = −0.03, *p* > .05) and MASC Social Anxiety subscale (*r* = 0.11, *p* > .05). All channels were re-referenced from Cz to the average reference. Baseline correction was conducted on each segment using the 100 ms pre-stimulus duration. Finally, an average of all participants’ trials of the same condition was calculated (positive word condition: *M* = 40.6; *SD* = 8.9, Range = 10–52; negative word condition: *M* = 40.5; *SD* = 8.8, Range = 15–52).

**Fig. 2.**
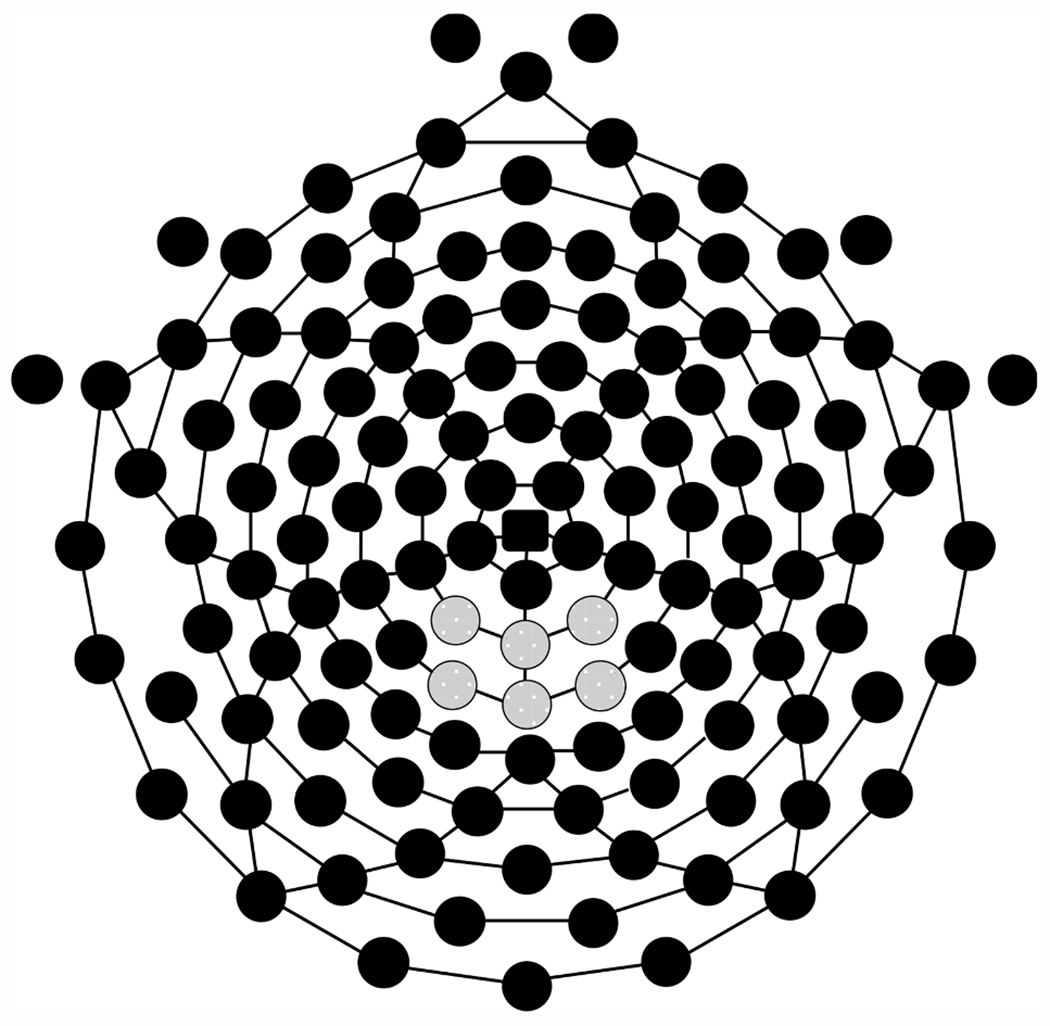
128-channel Geodesic sensor net sensor layout (Electrical Geodesics, Inc.) with electrodes capturing ERP components at the posterior regions specified in gray.

The posterior LPP site used to examine the averaged stimulus-locked ERPs was selected based on prior research (Auerbach et al., 2015; Waters and Tucker, 2013) and included electrodes 61, 62, 67, 72, 77, and 78 (Auerbach et al., 2016) (see Fig. 2). The posterior LPP was calculated as the mean amplitude between 600 ms-1200 ms word post-stimulus onset.

#### 2.4.2. Drift diffusion modeling (DDM)

**Fig. 3.**
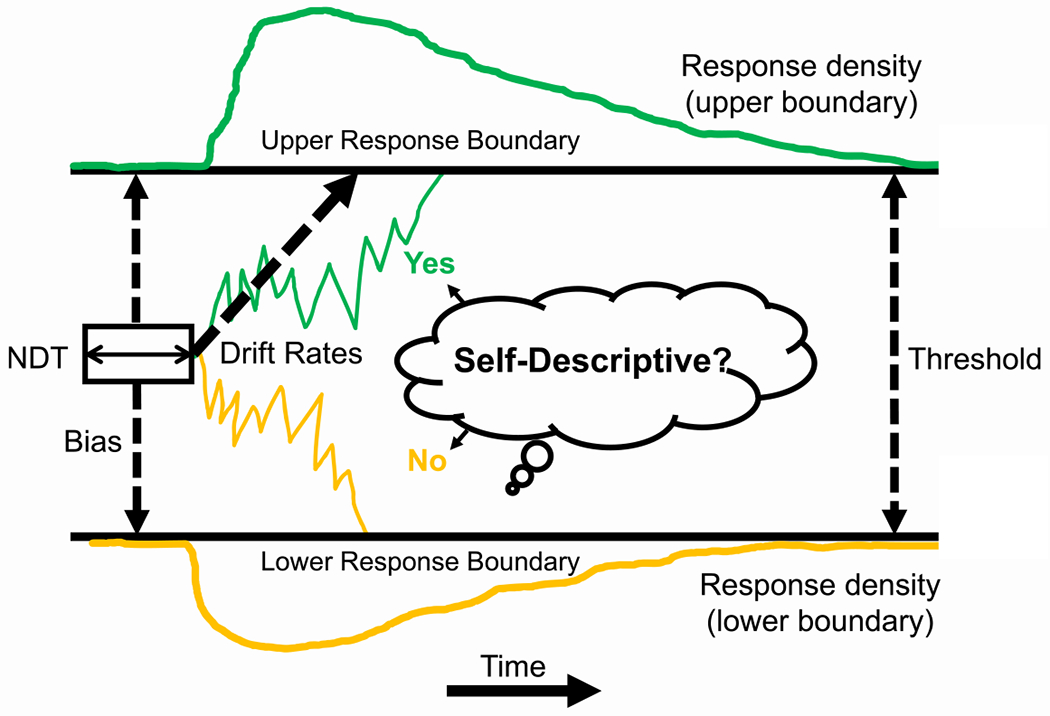
Simulated trajectories of the two drift-processes during the SRET (black and gray lines). Evidence is accumulated over time (x-axis) where the drift-rate per trial continues until it reaches one of two boundaries. The degree of separation is defined by threshold that, when crossed, a response is initiated (e.g., button press); an individual’s starting point along the y-axis is defined by the bias parameter. The solid arrow line in the beginning of the drift-processes indicates the non-decision time, NDT, where no accumulation happens. Upper (green) and lower (yellow) distributions indicate density plots for the two responses (yes, self-descriptive versus no, not self-descriptive). Although simulation data is depicted here, HDDM uses a closed-form likelihood function.

We examined youth SRET performance through drift diffusion modeling (Ratcliff, 1985; Ratcliff and Childers, 2015; Ratcliff and McKoon, 2008; Ratcliff and Rouder, 1998; Smith and Ratcliff, 2004; Stone, 1960); the standard for modeling response-time data from simple two-alternative forced choice decision-making tasks (Smith and Ratcliff, 2004) (see Fig. 3). We utilized the Hierarchical Drift Diffusion Modeling (HDDM 0.8.0) Python toolbox (Wiecki et al., 2013). Markov-chain Monte-Carlo was used to estimate posterior distributions of the drift-diffusion parameters, where trial type served as a factor with two levels (i.e., positive versus negative self-descriptive adjectives). HDDM user-defined models were created via Python script. Given the nature of the SRET, stimulus coding was used for modeling and all of our analyses. Specific parameters are thought to reflect rate of evidence accumulation (i.e., drift rate), amount of evidence required to make a decision (i.e., decision threshold), amount of evidence required to make one decision, inversely affecting the alternative (i.e., bias), and stimulus encoding and motor execution (i.e., non-decision time). Consistent with past research modeling the SRET (Dainer-Best et al., 2018), trials with reaction times under 200 ms were removed, as were trials at least three median absolute deviations above individual participant’s median reaction time (Leys et al., 2013).

For all models, 20,000 samples were generated from posteriors with the first 5000 serving as a burn-in, and every second sample was discarded as part of a thinning procedure. To formally test convergence, the Gelman-Rubin statistic (Gelman and Rubin, 1992) was calculated (10, 000 iterations, 1000 burn-in each) for each participant and found to be ≧ 1.1. Taken together with visual inspection of autocorrelation, trace, and histogram plots, there were no indications of problems with model convergence. We previously established a drift diffusion model allowing both drift rate and bias parameters to vary as a function of SRET conditions to be the best fit to youth task performance (Castagna et al., 2022). Therefore, six parameters were extracted for each participant to quantify performance: drift rate (for both positive- and negative-adjective trials), bias (for both positive- and negative-adjective trials), decision threshold, and non-decisional time. Statistical analyses focused on drift rate (processing efficiency) on the basis of past work that used a DMM framework to associate domain-general facets of anxiety with SRET performance (Castagna et al., 2022).

#### 2.4.3. Correlation & moderation analysis

**Fig. 4.**
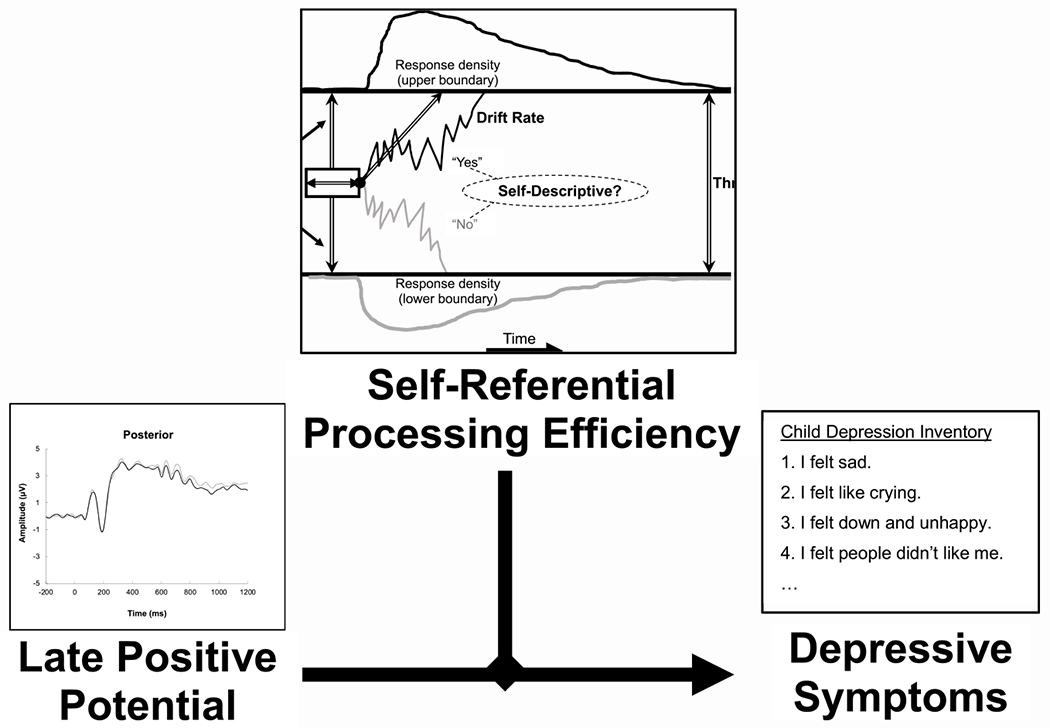
Moderation model examined depicting the relation between the posterior late positive potential response during the self-referential encoding task and youth depressive symptoms moderated by self-referential processing efficiency (drift rate).

First, we examined the relations among drift diffusion parameter estimates (i.e., drift rate, decision threshold, bias, nondecision time), posterior LPP (positive- and negative-adjective trials), major depressive symptoms (CDI total score), social anxiety (Social Anxiety MASC Subscale), sex, and age via Pearson correlations. Next, a single linear regression was utilized to conduct a moderation analysis (see Fig. 4). Our moderation analysis focused on the drift rate during the SRET, as this parameter has been found to be most strongly related to psychopathology (Dainer-Best et al., 2018) and is consistent with our previous work (Castagna et al., 2022). We calculated drift rate and posterior LPP difference scores based on positive and negative adjective SRET trials. Specifically, drift rate (and posterior LPP) during negative adjective trials was subtracted from positive adjective trials of the SRET to generate Δdrift rate and Δposterior LPP, respectively. Therefore, negative ends of the Δdrift rate spectrum reflect a tendency towards less efficient processing when determining if negative, relative to positive, adjectives are related to the self. This decision was made so these variables would reflect an individual’s *relative* difference in SRET processing efficiency (drift rate) and neural response (posterior LPP) (respectively) between positive and negative adjective trials, which also reduced the number of statistical comparisons needed to test the hypothesis.

A linear regression model was conducted to examine whether the relation between youth posterior LPP during the SRET and depressive symptoms is moderated by their processing efficacy (drift rate) when evaluating positive/negative adjectives. The model also included social anxiety symptoms as a covariate. All predictor variables were centered prior to analysis. Specifically, the moderation analysis predicted youth self-reported depressive symptoms, where Δdrift rate, Δposterior LPP, Δdrift rate x Δposterior LPP interaction, and social anxiety symptoms (covariate) served as predictors. Age was not included as covariate as it was found to be unrelated to depressive symptoms (*r* = 0.03, *p* > .05). Sex was not utilized as a covariate as it was unrelated to depression symptoms, t(105) = −0.91, *p* > .05. Correlational and regression analyses were conducted in R.

## 3. Results

### 3.1. Behavioral performance

During the SRET participants mean reaction time to positive adjectives was 0.41 s (SD = 0.12) and 0.45 s (SD = 0.18) to negative adjectives. In terms of endorsement, a mean of 37 (SD = 8.24) positive words were endorsed compared to a mean of 11 (SD = 9.65) negative adjectives.

### 3.2. Difference scores

At the group-level, youth in our community sample processed positive adjectives (*M* = 1.00, *SD* = 0.75) more efficiently than negative adjectives (*M* = −1.40, *SD* = 0.90) when making the decision if an adjective describes him/her. Negative ends of the Δdrift rate spectrum reflect a tendency towards less efficient processing when determining if negative, relative to positive, adjectives are related to the self. The posterior LPP amplitudes to negative adjectives (*M* = 2.71, *SD* = 3.12), compared to positive adjectives (*M* = 2.37, *SD* = 3.56) were statistically similar. Therefore, in terms of interpretability, positive ends of the Δposterior LPP dimension suggest a propensity towards larger posterior LPP amplitudes during negative, compared to positive, adjective trials of the SRET.

### 3.3. Correlation analyses

As shown in Table 1, drift rate (processing efficiency) during negative adjective trials showed a positive correlation with both depressive (*r* = 0.56, *p* < .001) and social anxiety symptoms (*r* = 0.41, *p* < .001). In contrast, the drift rate during positive adjective trials was inversely correlated with depressive (*r* = −0.43, *p* < .001) and social anxiety symptoms (*r* = −0.27, *p* = .005). Observed as a difference score, Δdrift rate (negative minus positive adjective SRET trials) showed a positive correlation with depressive (*r* = 0.55, *p* < .001) and social anxiety symptoms (*r* = 0.38, *p* < .001), as well as the Δposterior LPP (negative minus positive adjective SRET trials) (*r* = 0.24, *p* = .013). The Δposterior LPP was also negatively associated with youth depressive symptoms (*r* = −0.20, *p* = .39). In summary, elevated self-reported depression and social anxiety were associated with greater processing efficiency for negative adjectives and less efficiency for positive adjectives as well as reduced neural (posterior LPP) response to positive (relative to negative) adjectives.

### 3.4. Moderation analysis

**Fig. 5.**
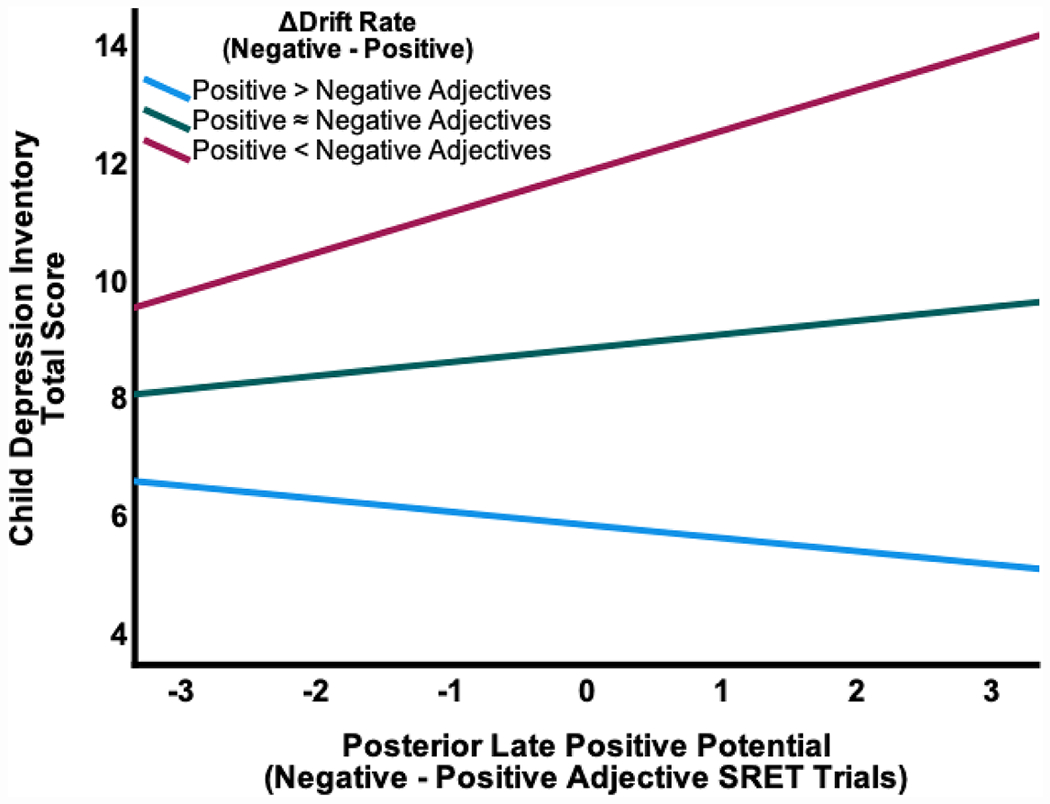
The relation between the difference score of the posterior late positive potential response during the self-referential encoding task and youth depressive symptoms moderated by the drift rate difference score. *Note*. Difference score = negative adjective trials minus positive adjective trials.

The overall model was significant, F(4, 105) = 25.05, *p* < .001, R^2^ = 0.50, Adjusted R^2^ =0.48, where social anxiety (β = 0.32, *t*(105) = 4.08, *p* < .001, CI_Lower_ = 1.03, CI_Upper_ = 2.97), Δdrift rate (α = 0.51, *t*(105) = 6.25, *p* < .001, CI_Lower_ = 2.13, CI_Upper_ = 4.11), Δposterior LPP (β = −0.35, *t*(105) = −3.37, *p* = .001, CI_Lower_ = −3.41, CI_Upper_ = −0.88), as well as the Δdrift rate by Δposterior LPP interaction (Δ = −0.34, *t*(105) = −3.29, *p* = .001, CI_Lower_ = −3.32, CI_upper_ = −0.82) were significant predictors of youth depressive symptoms. As shown in Fig. 5, more efficient processing of negative words during the SRET was associated with a positive relation between depressive symptoms and posterior LPP amplitude during negative (relative to positive) trials. In contrast, this relation was reversed among those with more efficient processing of positive words during the SRET.

## 4. Discussion

We found support for our hypothesis; youth SRET performance, decomposed via a drift-diffusion model, moderated the relation between posterior LPP during self-referential decision-making and youth depressive symptoms. Importantly, this relationship was robust when controlling for symptoms of social anxiety. Specifically, our results indicate that higher levels of depressive symptoms were associated with greater neural responsivity during negative adjective trials, but only in youth with a pattern of more efficient decision-making when determining if negative (but not positive) adjectives are self-descriptive. In fact, youth with a similar neural response (i.e., larger amplitudes in response to negative, compared to positive, adjectives), but that processed positive (compared to negative) adjectives more efficiently were more likely to display *lower* levels of self-reported depression. Processing efficiency (drift rate) during the SRET had a much weaker effect on the posterior LPP-depression relation when a greater posterior LPP response was observed during positive (relative to negative) adjective trials. Put differently, our findings suggest that depressive symptoms may be associated with a propensity towards differentially attending to negative self-referent information, potentially maintaining and/or exacerbating depressive symptoms, as this bias leads to a tendency for negative self-relevant information to become encoded.

It is important to interpret our findings with an understanding of the performance (Δdrift rate, processing efficiency) and posterior LPP (Δposterior LPP) responsivity difference scores; negative ends of the Δdrift rate spectrum broadly reflected a tendency towards less efficient processing when determining if negative adjectives were related to the self (when compared to their processing efficiency during positive adjective trials). Moreover, positive ends of the Δposterior LPP dimension suggest a propensity towards larger posterior LPP amplitudes during negative, compared to positive, adjective trials of the SRET. Overall, these findings provide a more nuanced view of the interplay between cognitive-affective neurophysiological processes and computationally informed self-referential processing that may contribute to our understanding of depressive symptomatology. Specifically, our results highlight the importance of interpreting the relation between neural correlates of self-referential processing in the context of youth behavioral performance. For instance, the SRET has been successful in assessing treatment-related improvement in self-views (Thurston et al., 2017), which, taken with our findings, suggest the posterior LLP during the SRET may be a novel target for intervention.

A large portion of past research has focused on self-referential processing in child and adolescent depression (Auerbach et al., 2015; Goldstein et al., 2015; Hayden et al., 2014, 2013, 2008; Kuiper and Derry, 1982; Liu et al., 2020; Mackrell et al., 2013). We previously provided support for the utility of behaviorally modeling the SRET. Specifically, we found the rate of evidence accumulation (i.e., drift rate) during negative self-referential processing was related to social anxiety and depression symptomatology above-and-beyond mean task performance (Castagna et al., 2022). An innovation of the present study, however, is to examine the relation between the neural correlates of self-referential processing and depressive symptoms from a drift diffusion framework in youth. Here, we focused on the posterior LPP, as it has been implicated as a potential neurophysiological indicator of depressive symptomatology in youth (Dickey et al., 2021; MacNamara et al., 2016; Waters and Tucker, 2016) and is a stable neural response during the self-referential processing task (SRET; Auerbach et al., 2016). Thus, the aim of the current study was to leverage computational modeling of the SRET to evaluate the unique relations among drift diffusion parameters of self-referential processing, posterior LPP responsivity, and youth self-reported symptoms of depression. Specifically, we were interested in answering the question: does the magnitude of the established relationship between youth neural response during the SRET (posterior LPP) and depressive symptoms vary as a function of their self-referential processing efficiency (drift-diffusion drift rate)?

Our findings build upon past research examining parietal LPP during the SRET in youth. Specifically, it has been shown that depressed youth (Auerbach et al., 2015) and youth at-risk for depression (Speed et al., 2016) have a greater parietal LPP response to negative (relative to positive) self-descriptions (Auerbach et al., 2015; Speed et al., 2016). Our results are consistent with past research in typically functioning youth, where similar parietal LPP amplitudes were reported in response to positive/negative adjectives (Auerbach et al., 2016). Behaviorally, our computational modeling findings are consistent with a previous study applying a drift-diffusion framework to SRET performance a sample of adults’ with and without remitted depression (Allison et al., 2021). Specifically, although both groups did not differ in their drift rate (processing efficiency) to positive adjectives, adults with remitted depression (compared to their typically functioning counterparts) tended to have slightly poorer processing efficacy (lower drift rate) when deciding if a negative (compared to positive) adjective was self-descriptive. Here, we found that a pattern of improved efficiency (larger drift rates) in processing negative adjectives (similar to those found in remitted depressed adults) predicted higher levels of depressive symptoms when youth also displayed greater neural responsivity (posterior LPP) during these same negative adjective trials.

In terms of neural processing, depressed (Auerbach et al., 2015) and at-risk (Speed et al., 2016) adolescents have demonstrated greater posterior LPP activity following negative (relative to positive) words over parietal-occipital regions (Auerbach et al., 2015 #939;Waters, 2013 #937). Similarly, individuals with remitted depression have shown diminished posterior LPP amplitudes to positive adjectives endorsed during the SRET (Allison et al., 2021). Although more research is needed, this appears to be consistent with our moderation findings; higher levels of youth depressive symptoms were associated with reduced posterior LPP amplitudes (and poorer processing efficiency) during positive adjective trials. The larger literature suggests that positive self-referential processing is reflected by the posterior LPP (Dainer-Best et al., 2017), which may be mediated by global emotional arousal (Sui and Humphreys, 2015). Although speculative, attenuated posterior LPP amplitudes may not only reflect blunted emotional arousal while processing positive self-referential stimuli in adults with remitted depression (Allison et al., 2021), but could also signify a neural profile of youth at-risk for experiencing elevated depressive symptoms (Auerbach et al., 2015; Speed et al., 2016). Indeed, past work has demonstrated that reduced emotional arousal during positive self-referential processing may inhibit reinforcement of positive self-schemas, thereby increasing an individual’s vulnerability for experiencing depressive symptoms in the future (Mennin and Fresco, 2013). Taking our findings together with past research, we suggest that depressive symptoms may be associated with a propensity towards differentially attending to negative self-referent information, potentially maintaining and/or exacerbating depressive symptoms as this bias leads to more negative self-relevant information to become encoded.

A couple of strengths of our study build upon this past work. First, we considered the potential confounding effect of comorbid social anxiety, as findings suggest that the posterior LPP response may be differentially related to symptoms of depression and anxiety (MacNamara et al., 2016; McLean et al., 2020; Sandre et al., 2019; Weinberg and Sandre, 2018). Second, we utilized a drift diffusion model of youth SRET performance, allowing for the extraction of parameters reflecting underlying latent self-referential cognitive processes (e.g., drift rate, processing efficiency). The latter aided in uncovering the moderation effects observed between youth parietal LPP and self-reported depressive symptoms.

### 4.1. Limitations & future directions

Limitations of the current study warrant consideration. We relied on a community sample. Although we speculate on how these results may fit with the larger depression literature, they can only be generalized to healthy youth. Participant psychiatric diagnostic status for initial study eligibility was also determined from parent self-report, which is not as valid as a structured clinical interview. Future work should include clinically depressed participants to further probe the relation between SRET performance and neural responsivity as indexed by the posterior LPP. In addition, our study focused on depressive symptomatology, while controlling for social anxiety; however, research would likely benefit from examining how these relations function in clinical anxious children and adolescents. Our study also relied on a relatively small, homogenous sample. It is notable that many of our primary results indicate a medium to large effect size. Replication in a larger, more diverse sample would greatly contribute to this area of research. Together with our findings and past work (Goldstein et al., 2015; Liu et al., 2020; Tracy et al., 2021), future research on self-referential processing would likely benefit from providing more attention to positive self-referential views as a protective factor against developing clinically significant depressive symptoms.

## 5. Conclusions

The current study addressed the question: does the magnitude of the established relation between youth neural response during the task (posterior LPP) and depressive symptoms vary as a function of their self-referential processing efficiency (drift rate)? After applying a drift diffusion computational model to youth performance on the self-referential encoding task (SRET), we found support for our hypothesis: higher levels of youth depressive symptoms were found in those with greater neural responsivity during negative adjective trials, but only in youth with a pattern of processing negative (relative to positive) adjectives more efficiently when deciding if the word is self-descriptive. Processing efficiency (drift rate) during the SRET had a much weaker effect on the posterior LPP-depression relation when a greater neural responsivity, as indexed by the posterior LPP, was observed in positive, compared to negative, adjective trials. Taken together with past research indicating that the SRET is sensitive to treatment-related improvements in one’s self-view (Thurston et al., 2017), our findings indicate that the posterior LLP during the SRET may serve as a novel target for intervention. In sum, our data extend the literature by highlighting the advantage of modeling the SRET in youth, while also highlighting the importance of interpreting the relation between neural correlates of self-referential processing in the context of youth behavioral performance when relating task-based behavior to depression symptoms in youth.

## Supplementary Material

1

## Figures and Tables

**Table 1:** Correlations among DDM parameters of SRET task performance, depression (CDI Total Score), social anxiety (MASC Social Anxiety subscale), posterior late positive potential, and demographics.

	1.	2.	3.	4.	5.	6.	7.	8.	9.	10.	11.	12.	13.	14.	*M*	*SD*
1. Drift Rate (Negative)	–														−1.40	.90
2. Drift Rate (Positive)	−0.64	–													1.00	.75
3. ΔDrift Rate	.92	−0.89	–												−0.2.40	1.49
4. Bias (Negative)	−0.03	.15	.09	–											.50	.02
5. Bias (Positive)	−0.38	.24	−0.35	−0.08	–										.52	.02
6. ΔBias	−0.23	.06	.17	−0.75	.72	–									−0.02	.03
7. Decision Threshold	.16	−0.17	−0.18	.05	−0.11	−0.11	–								1.07	.23
8. Non-decisional Time	.06	.03	−0.02	.02	.06	.03	−0.26	–							.16	.05
9. CDI Total Score	.56	−0.43	.55	−0.28	−0.27	.02	−0.12	.11	–						11.79	6.15
10. Social Anxiety	.41	−0.27	.38	−0.07	−0.31	−0.16	.01	.12	.55	–					10.86	6.21
11. Posterior LPP (Neg)	−0.12	−0.02	.06	.01	.14	.09	.02	−0.05	−0.05	−0.13	–				2.71	3.12
12. Posterior LPP (Pos)	−0.10	.06	.09	−0.04	.07	.07	.12	−0.06	.05	.00	.78	–			2.37	3.56
13. ΔPosterior LPP	.00	−0.12	.24	.08	.09	.00	.14	−0.18	−0.20	−0.15	.15	−0.50	–		.34	2.23
14. Child Age	.03	.06	.01	.11	−0.02	−0.09	−0.21	.19	.01	.03	−0.28	−0.19	−0.08	–	14.49	1.70
15. Child Sex	.18	.07	−0.07	−0.14	−0.08	.04	.02	.37	.09	.20	−0.17	−0.09	−0.10	.04	1.47	.50

*Note*. bold = *p* < .05; sex coding: boy = 1, girl = 2; CDI = Child Depression Inventory; posterior LPP = posterior late positive potential.

## Appendix A

Words used in the Self-Referential Processing Task

**Table:**

Negative Words	Positive Words
Rejected	bright
Shamed	untroubled
Displeased	lively
Guilty	masterful
Lost	hopeful
Crushed	satisfied
Burdened	vigorous
Afraid	alive
Defeated	capable
Rude	carefree
Dreadful	dignified
Angry	loyal
Brutal	admired
Useless	grateful
Stupid	happy
Morbid	bold
Anguished	elated
Upset	festive
Insecure	adorable
Frustrated	gentle
Insane	proud
Alone	lucky
Disgusted	joyful
Bored	jolly
Troubled	inspired
Unhappy	devoted
Terrified	friendly
Fearful	wise
Terrible	famous
Lonely	thoughtful
Sinful	confident
Obnoxious	terrific
Helpless	silly
Disloyal	useful
Cruel	honest
Violent	engaged
Scared	cute
Hostile	outstanding
Depressed	beautiful
Distressed	helpful
Gloomy	cheerful
Cranky	pleasant
Moody	brave
Impatient	optimistic
Lazy	funny
Nervous	polite
Harsh	peaceful
Ashamed	curious
Grumpy	daring
Weak	calm
Jealous	loving
Worried	surprised
