# Supplementary material for: Catch the drift: Depressive symptoms track neural response during more efficient decision-making for negative self-referents
Source: J Affect Disord Rep. Author manuscript; Available in PMC 2023 Jul 1. (PMC10310306; doi:10.1016/j.jadr.2023.100593)
Supplement: 1 [file NIHMS1909857-supplement-1.docx]

**Supplemental Materials**

**Figure 1.** Posterior plots for the group mean of the drift rate (top left), decision threshold (top right), bias (bottom left), and nondecision time (bottom right) parameters; posterior trace (upper left in lay), autocorrelation (lower left in lay), and marginal posterior histogram (right in lay), where the solid black line denotes posterior mean and dotted black line denotes 2.5 and 97.5% percentiles, are provided.

**Table 1.** Convergence statistics of hierarchical drift diffusion models using stimulus coding.

|  | **DIC** |
| --- | --- |
| Null Model without Bias | 2852 |
| *Null Model with Bias* | *2841* |
| 1) Drift Rate Split Model | 702 |
| 2) Threshold Split Model | 2863 |
| 3) Bias Split Model | 2827 |
| 4) Drift Rate & Threshold Split Model | 692 |
| 5) Threshold & Bias Split Model | 2850 |
| **6) Drift Rate & Bias Split Model** | **654** |
| 7) Drift Rate, Threshold, & Bias Split Model | 661 |

*Note.* DIC = deviance information criterion; italics = best fitting null model; bold = best fitting model.

**Figure 2.** Posterior probability of drift rate group means for positive (blue) and negative (red) adjective trials during the SRET (A); posterior probability of bias group means during the SRET (B); within-subject model group mean posterior probability of drift rate during the SRET (C); within-subject model group mean posterior probability of bias during the SRET (D).

A)

B)

C)

D)
